# Identification of the Biotransformation Pathways of a Potential Oral Male Contraceptive, 11β-Methyl-19-Nortestosterone (11β-MNT) and Its Prodrugs: An In Vitro Study Highlights the Contribution of Polymorphic Intestinal UGT2B17

**DOI:** 10.3390/pharmaceutics16081032

**Published:** 2024-08-02

**Authors:** Namrata Bachhav, Dilip Kumar Singh, Diana L. Blithe, Min S. Lee, Bhagwat Prasad

**Affiliations:** 1 College of Pharmacy and Pharmaceutical Sciences, Washington State University (WSU), Spokane, WA 99202, USA; 2 Contraceptive Development Program, Division of Population Health Research, Eunice Kennedy Shriver National Institute of Child Health and Human Development, National Institutes of Health, 1 Center Dr, Bethesda, MD 20892, USA

**Keywords:** biotransformation, 11β-Methyl-19-nortestosterone, 11β-MNT, 11β-MNTDC, UGT2B17, oral male contraceptive, metabolite identification, LC-MS, MetID

## Abstract

11β-Methyl-19-nortestosterone dodecylcarbonate (11β-MNTDC) is a prodrug of 11β-MNT and is being considered as a promising male oral contraceptive candidate in clinical development. However, the oral administration of 11β-MNTDC exhibits an ~200-fold lower serum concentration of 11β-MNT compared to 11β-MNTDC, resulting in the poor bioavailability of 11β-MNT. To elucidate the role of the first-pass metabolism of 11β-MNT in its poor bioavailability, we determined the biotransformation products of 11β-MNT and its prodrugs in human in vitro models. 11β-MNT and its two prodrugs 11β-MNTDC and 11β-MNT undecanoate (11β-MNTU) were incubated in cryopreserved human hepatocytes (HHs) and subjected to liquid chromatography–high resolution tandem mass spectrometry analysis, which identified ten 11β-MNT biotransformation products with dehydrogenated and glucuronidation (11β-MNTG) metabolites being the major metabolites. However, 11β-MNTG formation is highly variable and prevalent in human intestinal S9 fractions. A reaction phenotyping study of 11β-MNT using thirteen recombinant UDP-glucuronosyltransferase (UGT) enzymes confirmed the major role of UGT2B17 in 11β-MNTG formation. This was further supported by a strong correlation (R^2^ > 0.78) between 11β-MNTG and UGT2B17 abundance in human intestinal microsomes, human liver microsomes, and HH systems. These results suggest that 11β-MNT and its prodrugs are rapidly metabolized to 11β-MNTG by the highly polymorphic intestinal UGT2B17, which may explain the poor and variable bioavailability of the drug.

## 1. Introduction

**Figure 1:**
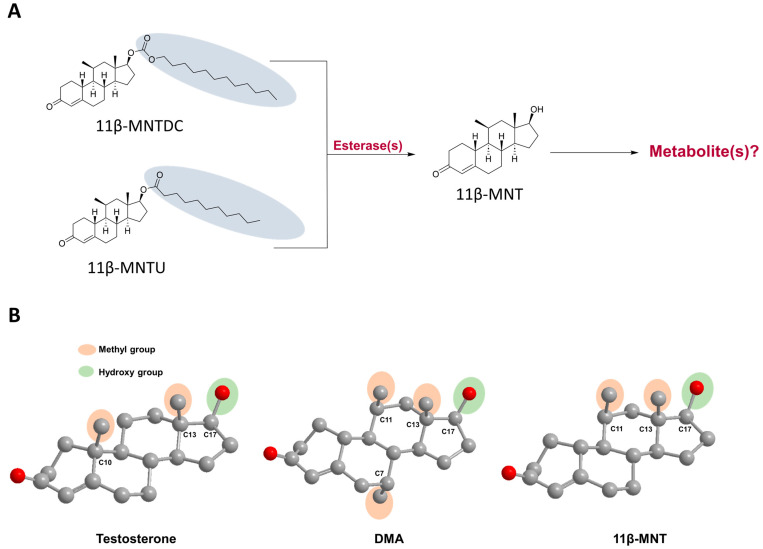
11β-Methyl-19-nortestosterone dodecylcarbonate (11β-MNTDC) and 11β-Methyl-19-nortestosterone undecanoate (11β-MNTU) are prodrugs metabolized by esterases into their active metabolite, 11β-Methyl-19-nortestosterone (11β-MNT) (**A**). Structural similarities amongst testosterone, dimethandrolone (DMA), and 11β-MNT (**B**). Red spheres in (**B**) indicate oxygen atom.

Approximately 121 million unplanned pregnancies representing 6.4% of the total number of women aged 14 to 49, occurred annually across the globe between 2015 and 2019 [1], and approximately 50% of these pregnancies resulted in abortions. Although a range of effective contraception methods are available for women such as birth control tablets to intrauterine devices, millions of women worldwide have unsafe abortions each year. The lack of male contraceptive methods in family planning is considered one of the key reasons for this challenge [2]. The imperative need for oral male contraceptives has become a subject of increased interest as society progresses toward greater gender equality and the demand for alternatives to female contraceptives increases. 11β-Methyl-19-nortestosterone dodecylcarbonate (11β-MNTDC) is a promising investigational male contraceptive drug under clinical development. 11β-MNTDC is a novel synthetic androgen that binds to both androgen and progesterone receptors and is thought to potentially serve as a single-agent male contraceptive [3]. 11β-MNTDC is converted in vivo to the active metabolite, 11β-Methyl-19-nortestosterone (11β-MNT) [4]; however, the subsequent metabolism of the active drug and the site of metabolism (intestine versus liver) are still unknown (Figure 1A).

We recently identified that dimethandrolone (DMA), a structural analog of 11β-MNT, is rapidly glucuronidated by one of the most polymorphic enzymes, UDP-glucuronosyltransferase 2B17 (UGT2B17), potentially contributing to its poor and variable first-pass metabolism [5]. UGT2B17 also plays a critical role in the glucuronidation and first-pass elimination of testosterone [6]. Due to the structural similarities of 11β-MNT, DMA, and testosterone (Figure 1B), we hypothesized that the poor and variable bioavailability of 11β-MNT [7] is also linked to its extensive intestinal and hepatic first-pass metabolism by UGT2B17. Glucuronidation is a crucial metabolic step that involves the conjugation of a drug with glucuronic acid through UGT enzyme(s), making it more water-soluble to facilitate elimination [8]. However, the role of the UGTs in relation to 11β-MNT is not well characterized.

An in-depth understanding of 11β-MNT’s biotransformation products and the enzymes involved in its metabolism will be crucial for interpreting or predicting its clinical pharmacokinetics (PK), poor and variable bioavailability, and potential interactions with other pharmaceutical drugs. Additionally, the exploration of metabolite identification is useful in providing insights into potential structural modifications for the development of more bioavailable analogs of 11β-MNT. In this study, we utilized human hepatocytes (HHs) as well as intestinal and hepatic S9 fractions to understand the metabolic fate of 11β-MNT and the prodrugs, 11β-MNTDC and 11β-MNT undecanoate (11β-MNTU). A novel optimized metabolomics-based strategy was leveraged in a high throughput manner to detect and identify metabolites in the in vitro incubates. In addition, to identify which UGT isoform(s) is responsible for the glucuronidation of 11β-MNT, we utilized thirteen specific recombinant UGT enzymes in an in vitro reaction phenotyping study. 

Understanding the metabolic fates of 11β-MNT and its prodrugs is also crucial for developing a protocol for clinical trials and their effective outcomes. This entails taking into consideration the impact of polymorphic enzymes on the metabolism of these compounds, which in turn affects their oral bioavailability. 

## 2. Materials and Methods

The 11β-MNT, 11β-MNTDC, 11β-MNTU, and deuterated 11β-MNT (11β-MNT-d6) were provided by the Eunice Kennedy Shriver National Institute of Child Health and Human Development (NICHD, Bethesda, MD, USA) through SRI International (Menlo Park, CA, USA). The cryopreserved adult HHs (*n* = 4), hepatocyte thawing (HT) media, and hepatocyte incubation (HI) media were provided by BioIVT (Westbury, NY, USA; Table S1). The human intestinal and liver S9 fractions were purchased from XenoTech (Kansas City, KS, USA). The individual human intestinal microsomes (HIMs) were procured from BioIVT (Westbury, NY, USA), whereas the individual human liver microsomes (HLMs) were in-house generated and available in our laboratory from another study. The recombinant human UGT enzymes (rUGTs) were purchased from Corning Life Sciences (Riverfront, NY, USA). The alamethicin, UDP-glucuronic acid (UDPGA), magnesium chloride (MgCl_2_), and mono- and di-basic potassium phosphate were purchased from Sigma-Aldrich (St. Louis, MO, USA). The deuterated testosterone glucuronide (TG-d3) standard was obtained from Cerilliant Corporation (Round Rock, TX, USA).

### 2.1. 11β-MNT, 11β-MNTDC, and 11β-MNTU Incubation in Human Hepatocytes, Human Intestinal, and Liver S9 Fractions

11β-MNT, 11β-MNTDC, and 11β-MNTU were incubated in a HH suspension to generate primary and secondary metabolites for metabolite identification. Adult HHs (*n* = 4; two high expressors and two null expressors of UGT2B17), were thawed in a water bath at 37 °C and carefully poured into a 10 mL conical tube containing a pre-warmed HT medium. The cell suspension was gently mixed by gently inverting the tube and the medium was removed by centrifugation at 50× *g* for 5 min at room temperature. The supernatant was removed and the pellet containing the HHs was mixed with 3 mL of pre-warmed HT medium. The cell counting was performed using the trypan-blue exclusion technique using an Auto T4 cellometer (Nexcelom Bioscience, Lawrence, MA, USA). A solution containing 1 million cells per mL was prepared in a HI medium and 300 μL (0.3 × 10^6^ cells) of the HH suspension was incubated for 15 min after seeding onto a 24-well plate. 11β-MNT (10 µM and 50 µM), 11β-MNTDC (10 µM), and 11β-MNTU (10 µM) were added to the HH suspension for a 120 min incubation at 37 °C. The reaction was quenched with the addition of 0.6 mL of ice-cold acetonitrile containing an internal standard (TG-d3, 100 ng/mL). The reaction mixture was vortex-mixed for 5 min and centrifuged at 10,000× *g* for 10 min at 4 °C. The supernatant (50 µL) was diluted with 100 µL of 0.1% formic acid and transferred to a LC vial for analysis. The same four lots of HH samples treated with the vehicle alone (without the drugs) and processed using the same protocol were considered as control samples for metabolomics analysis. 

11β-MNT, 11β-MNTDC, and 11β-MNTU were also incubated in the human intestine and liver S9 fractions using optimized conditions to characterize the tissue-specific metabolite formation. The S9 protein (10 µg) was added to a buffer solution comprising 100 mM potassium phosphate (pH 7.4), 5 mM MgCl_2_, 0.01% bovine serum albumin, and 0.1 mg/mL of alamethicin (pore-forming agent). After a 15 min pre-treatment on ice, the reaction was initiated by adding 11β-MNT, 11β-MNTDC, and 11β-MNTU (50 µM each) and 2.5 mM UDPGA. The mixture was incubated at 37 °C for 30 min with a gentle shaking at 300 rpm. The reaction was stopped by adding 150 µL of ice-cold acetonitrile containing TG-d3 as an internal standard. After centrifuging the samples at 10,000× *g* for five minutes at a temperature of 4 °C, the supernatant was transferred to a LC vial for analysis. 

### 2.2. Characterization of UGT Isoforms Involved in the Glucuronidation of 11β-MNT

Thirteen recombinant human UGT enzymes (rUGT1A1, rUGT1A3, rUGT1A4, rUGT1A6, rUGT1A7, rUGT1A8, rUGT1A9, rUGT1A10, rUGT2B4, rUGT2B7, rUGT2B10, rUGT2B15, and rUGT2B17) were used to identify the isoforms involved in the glucuronidation of 11β-MNT. For each rUGT isoform, the enzyme (10 µg) was added to the incubation buffer (total volume, 100 µL) comprising potassium phosphate (100 mM, pH 7.4), bovine serum albumin (0.01%), MgCl_2_ (5.0 mM), and alamethicin (0.1 mg/mL). Following a 15 min pre-treatment on ice, the reaction was initiated by adding 11β-MNT and UDPGA at final concentrations of 50 µM and 2.5 mM, respectively. The incubation was carried out for 45 min at 37 °C in a shaking water bath (300 rpm) in triplicate. The reaction was stopped by adding 150 µL of ice-cold acetonitrile containing TG-d3 as the internal standard. After centrifuging the sample at 10,000× *g* for 5 min at 4 °C, the supernatant was transferred into a LC vial for analysis. 

### 2.3. Tissue-Specific 11β-Methyl-19-Nortestosterone-Glucuronide (11β-MNTG) Formation and UGT2B17 Abundance in Human Intestinal Microsomes (HIMs), and Human Liver Microsomes (HLMs)

The individual HIMs (*n* = 12) [9] and individual HLMs (*n* = 23) [10] previously characterized for protein abundance using quantitative proteomics were used to investigate the correlation between 11β-MNTG formation and UGT2B17 abundance. To incubate HIMs and HLMs, 10 µg of protein was added to 100 µL of buffer containing potassium phosphate (100 mM, pH 7.4), bovine serum albumin (0.01%), MgCl_2_ (5.0 mM), and alamethicin (0.1 mg/mL). After a 15 min pre-treatment on ice, the reaction was started by adding 11β-MNT and UDPGA at final concentrations of 50 µM and 2.5 mM, respectively. Incubation was performed in triplicate for 45 min at 37 °C in a shaking water bath (300 rpm). The reaction was halted with the addition of 150 µL of ice-cold acetonitrile containing 11β-MNT-d6 as an internal standard. After centrifugation at 10,000× *g* for 5 min at 4 °C, the supernatant was transferred to a LC vial for analysis.

### 2.4. LC-MS/MS Conditions for Acquisition of High-Resolution MS Data for Metabolite Identification in Human Hepatocytes, Human Intestinal, and Liver S9 Fractions

The high-resolution MS data were acquired using nano-LC coupled with a Q Exactive Orbitrap HF (Thermo Scientific, San Jose, CA, USA) in both full-MS and data-independent acquisition (DIA) modes using positive polarity. The metabolites were separated using a C18 column (25 cm × 50 µm, particle size 2 µm, and 100 Å) (Thermo Scientific, Waltham, MA, USA). The flow rate was set to 300 nL/min and a 1 µL sample was injected for analysis using the following mobile phase: A. water with 0.1% formic acid and B. 80% acetonitrile with 0.1% formic acid. The gradient program used for analysis was as follows: 0–1 min (30% B), 1–9 min (30 → 60% B), 9–14 min (60 → 80% B), 14–20 min (80% B), 20–25 min (80 → 100% B), and 25–45 min (100% B). Since 11β-MNTDC and 11β-MNTU are more lipophilic, the last step of the gradient was extended to 115 min for the analysis of incubations containing the prodrugs, i.e., 25–115 min (100% B). The gradient program for analysis of the S9 samples was as follows: 0–1 min (5% B), 1–4 min (5–50% B), 4–10 min (50–80% B), 10–15 min (80–100% B), and 20–30 min (100% B). 

### 2.5. Untargeted Metabolomics of 11β-MNT in Human Hepatocytes 

The acquired data were processed to identify metabolites using an optimized XCMS-based (Scripps Center for Metabolomics, La Jolla, CA, USA, version 3.7.1), untargeted metabolomics approach (Table S2). Briefly, untreated (control) and treated HH samples were initially compared using XCMS Online to identify the significantly higher *m*/*z* features (*p*-value < 0.05 and fold differences > 5-folds) in the treatment group. The significantly increased features were screened using a *m*/*z* range of 200–700 and by employing a mass defect filtration (MDF) criterion of −150 to +150 mDa [11]. These features were then matched and compared to the probable theoretical metabolites generated using a previously established approach. Furthermore, metabolites were identified using metabolic processes with a mass error < 2 ppm.

### 2.6. LC-MS/MS Conditions for Quantification of 11β-MNT Glucuronide (11β-MNTG) in Reaction Phenotyping Assay, HIMs, and HLMs 

An M-class microflow Waters UPLC system coupled with a Waters Xevo TQ-XS MS (Waters, Milford, MA, USA) equipped with a standard electrospray ionization source was used for the detection and quantification of 11β-MNT glucuronide in rUGT incubations, HIMs, and HLMs. For the reaction phenotyping assay, HIM, and HLM incubations, the mobile phase composed of two solvents: (A) 0.1% formic acid in water and (B) 0.1% formic acid in acetonitrile, was run using the following gradient program (reaction phenotyping assay): 0–0.5 min (10% B), 0.5–3.0 min (10 → 75% B), 3.0–6.0 min (75 → 95% B), 6.0–6.1 min (95 → 10% B), and 6.1–8.0 min (10% B), and the gradient program (HIM and HLM incubations): 0–0.3 min (60% B), 0.3–1.5 min (60 → 98% B), 1.5–6.6 min (98 → 98% B), 6.6–6.9 min (98 → 60% B), and 6.9–9.8 min (60% B). An Acquity UPLC^®^ HSS T3 (1.8 µm, 1 mm × 100 mm) column was used to separate the analytes. The LC conditions were set at a flow rate of 50 µL/min and the injection volume was 1 µL.

The multiple reaction monitoring (MRM) parameters for the quantification of 11β-MNT glucuronide in the rUGT incubations included an optimized cone voltage of 25 V, transitions of 11β-MNTG (*m*/*z* 465.1 → *m*/*z* 97.1 and *m*/*z* 109.1 at CE 40 eV; *m*/*z* 465.1 → *m*/*z* 271.2 at CE 25 eV; *m*/*z* 465.1 → *m*/*z* 289.2 at CE 22 eV; and *m*/*z* 465.1 → *m*/*z* 465.1 at CE 5 eV) and TG-d3 (*m*/*z* 468.2 → *m*/*z* 97.1 and *m*/*z* 109.1 at CE 40 eV; *m*/*z* 468.2 → *m*/*z* 256.2, *m*/*z* 274.2, and *m*/*z* 292.2 at CE 30 eV) in the positive ion mode. For the quantification of 11β-MNTG in HIMs and HLMs, MRM transitions for 11β-MNT (*m*/*z* 289.2 → *m*/*z* 97.1 and *m*/*z* 109.1 at CE 40 eV; *m*/*z* 289.2 → *m*/*z* 253.2 at CE 25 eV; *m*/*z* 289.2 → *m*/*z* 271.2 at CE 22 eV; and *m*/*z* 289.2 → *m*/*z* 289.2 at CE 5 eV), 11β-MNTG (*m*/*z* 465.1 → *m*/*z* 97.1 and *m*/*z* 109.1 at CE 40 eV; *m*/*z* 465.1 → *m*/*z* 271.2 at CE 25 eV; *m*/*z* 465.1 → *m*/*z* 289.2 at CE 22 eV; and *m*/*z* 465.1 → *m*/*z* 465.1 at CE 5 eV) and 11β-MNT-d6 (*m*/*z* 295.2 → *m*/*z* 97.1 and *m*/*z* 109.1 at CE 40 eV; *m*/*z* 295.2 → *m*/*z* 259.2 and *m*/*z* 277.2 at CE 25 eV; and *m*/*z* 295.2 → *m*/*z* 295.2 at CE 5 eV) in the positive ion mode.

## 3. Results

### 3.1. Identification of Biotransformation Products of 11β-MNT in Human Hepatocytes

**Figure 2:**
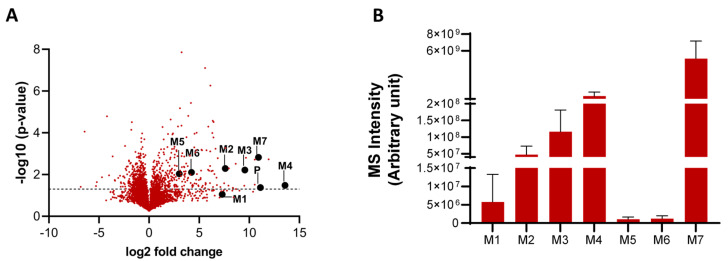
Volcano plot showing fold-differences and *p*-values of *m*/*z* features detected in a 2 h incubation of human hepatocytes with 11β-MNT as compared to the untreated (control) samples (**A**). P represents the parent active metabolite (11β-MNT); M1 and M3–M7, six identified metabolites; M2, four potential structural isomers. The black dotted line indicates −log10 (*p*-value, i.e., 1.33). Relative MS intensity of 11β-MNT metabolites in human hepatocyte incubation (**B**).

The analysis of untargeted high-resolution mass spectrometry (HRMS) data using XCMS Online software (version 3.7.1) revealed 7414 total mass features (*m*/*z* values) in the HH incubation of the high concentration (50 µM) of 11β-MNT (Table S1). Screening of the data by removing peaks with intensity < 300,000 and fold-increases (11β-MNT treated versus control) below 5-folds, provided a list of 231 features. Further screening of the potential metabolites (i.e., *m*/*z* range of 200–700 and MDF, −150 to +150 mDa) yielded a total of 167 remaining features. These 167 features were analyzed for probable metabolites using an in-house theoretical metabolite generator with an acceptance criterion of 2 ppm accuracy, which predicted 23 mass features (probable metabolites including their corresponding isotopic masses). Out of these, ten metabolites were identified based on whether the metabolite reaction is possible for the 11β-MNT structure. The volcano plot illustrates the parent and ten identified metabolites among a total of 7414 detected features (Figure 2A). Amongst these, M4 and M7 were the major metabolites with relative MS intensities of 12% and 85%, respectively (Figure 2B). Eight other metabolites (M1, M2a, M2b, M2c, M2d, M3, M5, and M6) were <2% in terms of relative MS intensities (Figure 2B). 

**Figure 3:**
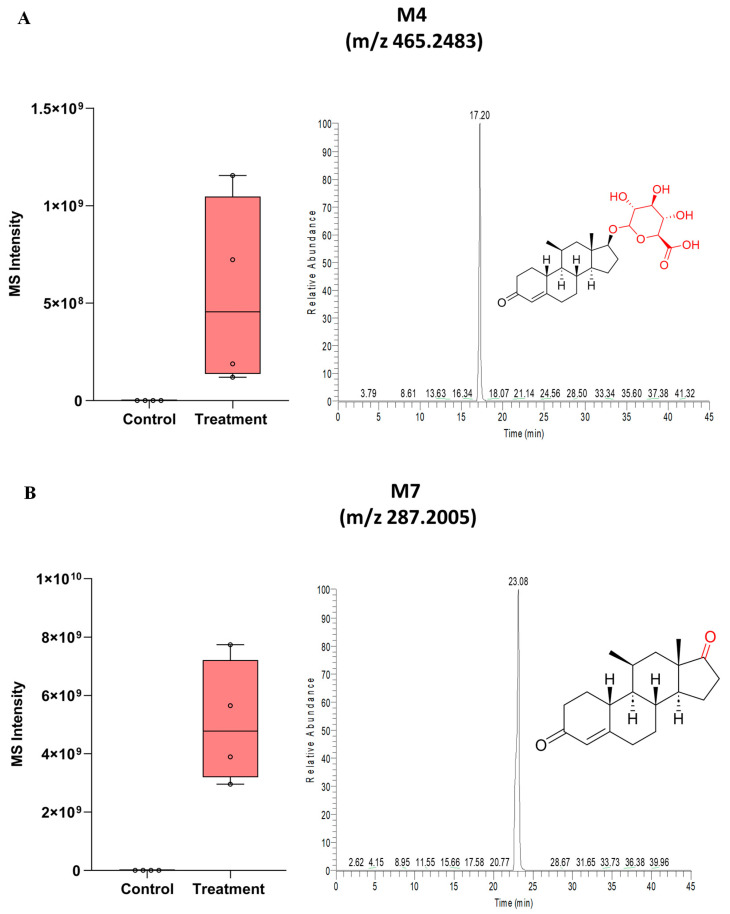
Box and Whisker plot representing 11β-MNT and its major metabolites, M4, i.e., 11β-MNTG (**A**) and M7 (**B**) showing signal in the treatment (50 µM of 11β-MNT) compared to the untreated group in human hepatocytes.

The signal intensities of all ten metabolites in the HH incubations (untreated versus 11β-MNT-treated) as well as their predicted structures are shown in Figure 3 and Supplementary Figure S1. The predicted metabolites of 11β-MNT in HH incubations were as follows: (i) M1: S-glutathione conjugate (addition of 305.0682 Da), (ii) M2a–d (M2a, M2b, M2c, and M2d): hydroxylated 11β-MNT (addition of 15.9949 Da), (iii) M3: dehydrogened and hydroxylated 11β-MNT (addition of 13.9792 Da), (iv) M4: 11β-MNT glucuronide or 11β-MNTG (addition of 176.0321 Da), (v) M5: glucosylated 11β-MNT (addition of 162.0528 Da), (vi) M6: based on the biotransformation pathway of the structural analog, testosterone, M6 is either androsterone glucuronide or its isomeric product (addition of 178.0478 Da), and (vii) M7: dehydrogenated 11β-MNT (removal of 2.0157 Da). M2 showed four distinct peaks (a–d) indicating four potential hydroxylated forms of 11β-MNT. Among the ten identified metabolites, M4 (11β-MNTG) and M7 were the major metabolites (Figure 3A,B), whereas all the other metabolites were minor, and thus not considered for further characterization.

**Figure 4:**
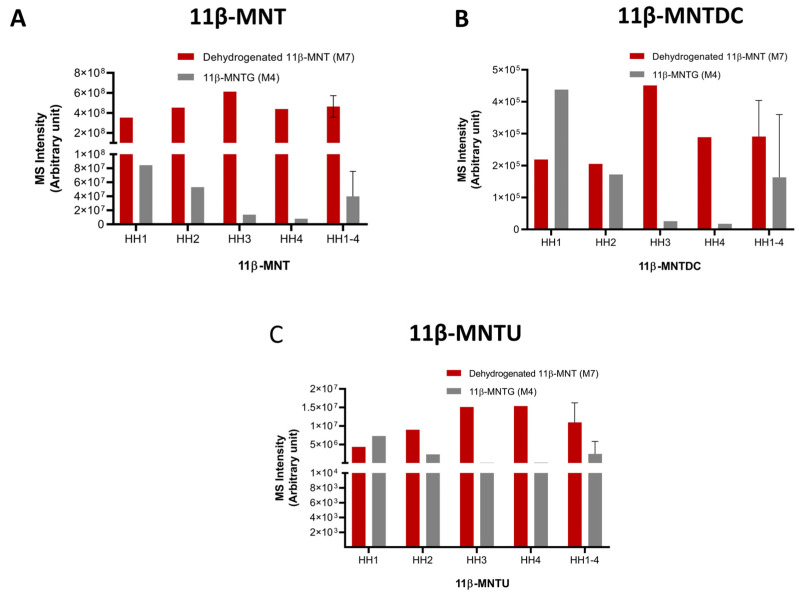
The levels of dehydrogenated 11β-MNT and 11β-MNTG formed in human hepatocyte incubations of 50 µM 11β-MNT (**A**) and 10 µM 11β-MNTU (**B**), and 11β-MNTDC (**C**). HH1–4: (Average of all four hepatocytes with SD). HH1 and HH2 represent UGT2B17 high expressors, whereas HH3 and HH4 are the null expressors of UGT2B17.

The HRMS data of the putative metabolites along with their chemical formulae, theoretical *m*/*z* mass errors, and retention times are provided in Table S3. The structural assignments of ten identified metabolites were further supported using their unique MS/MS fragmentation patterns (Figure S2, Tables S3 and S4). For example, M4 was confirmed by the presence of the unique neutral loss of 176 Da (glucuronide moiety). Compared to 11β-MNT (50 µM) (Figure 4A), only M4 and M7 were detected in the untargeted analysis of HH incubations at lower incubation concentrations (10 µM) of 11β-MNTDC (Figure 4B) and 11β-MNTU (Figure 4C). 

### 3.2. Formation of Biotransformation Products of 11β-MNT in Human Intestinal and Liver S9 Fractions

**Figure 5:**
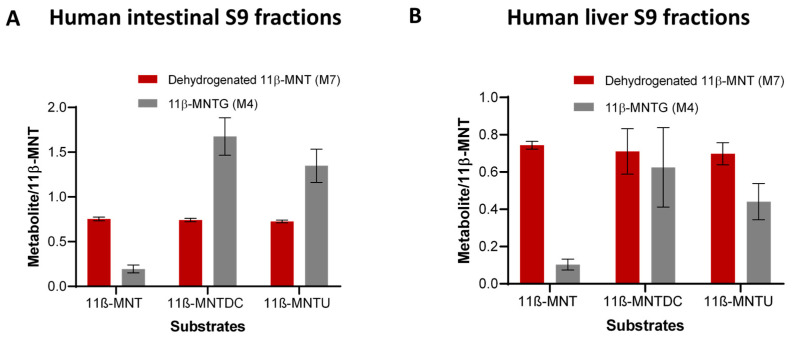
Metabolite/11β-MNT ratio of dehydrogenated 11β-MNT and 11β-MNTG in the pooled human intestinal (**A**) and liver (**B**) S9 fraction incubations with 50 µM of 11β-MNT, 11β-MNTDC, and 11β-MNTU.

Only the dehydrogenation (M7) and glucuronidation (M4) products were detected when 11β-MNT, 11β-MNTDC, and 11β-MNTU were incubated in the human intestine (Figure 5A) and liver (Figure 5B) S9 fractions. The relative formation of these two metabolites was comparable between human liver S9 fractions and HH incubations (Figure 4A–C) with dehydrogenated metabolites being the prominent metabolic product. However, the formation of 11β-MNTG was significantly greater in the human intestinal S9 fractions when compared to the formation of dehydrogenated 11β-MNT. 

### 3.3. Interindividual Variability in the Major Metabolic Pathways of 11β-MNT in Human Hepatocytes 

Although the levels of dehydrogenated 11β-MNT were consistent with a fold difference of ~2.5-fold (Figure 4B,C), 11β-MNTG was highly variable with an ~10-fold variability in HH incubations of the null versus high expressors of UGT2B17 (Figure 4A). 

### 3.4. Characterization of UGT Isoforms Involved in 11β-MNT Glucuronidation

**Figure 6:**
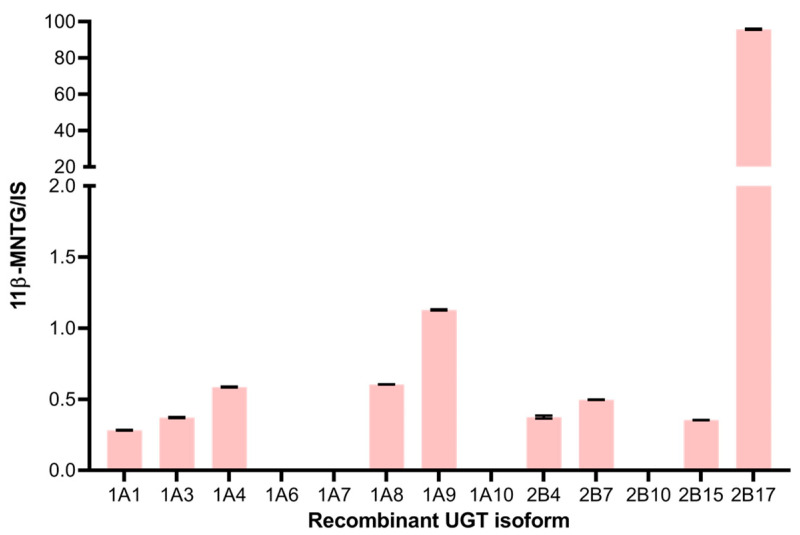
Normalized 11β-MNTG levels (with internal standard) detected in the incubation of individual recombinant UGT isoforms. SD represents triplicate analysis.

The intestinal S9 data were further complemented by the UGT reaction phenotyping assay. Out of the thirteen tested recombinant human UGTs, rUGT2B17 exhibited a major role in the conversion of 11β-MNT to 11β-MNTG (Figure 6). Although 11β-MNTG was also detected in rUGT1A4, rUGT1A8, rUGT1A9, and rUGT2B7 incubations, the relative intensity of the metabolite formed was <1% when compared to the rUGT2B17 incubation (Figure 6). 

### 3.5. Correlation between 11β-Methyl-19-Nortestosterone-Glucuronide (11β-MNTG) Formation and UGT2B17 Abundance in HIMs, HLMs, and HHs

**Figure 7:**
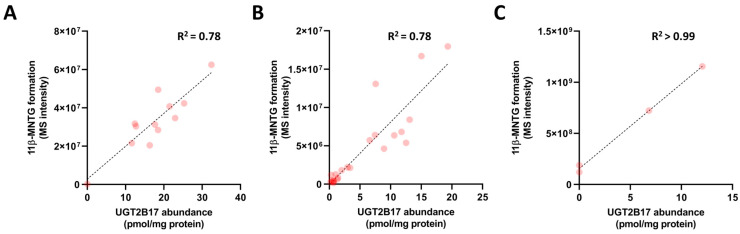
Correlation of UGT2B17 abundance and relative 11β-MNTG levels in HIMs (**A**), HLMs (**B**), and human hepatocytes (**C**).

To further confirm the role of UGT2B17 in 11β-MNTG formation, the correlation between the metabolite formation and UGT2B17 abundance was studied in individual HIM, HLM, and HH incubations. A good correlation of R^2^ > 0.78 was observed between UGT2B17 abundance and 11β-MNTG in HIMs (Figure 7A) and HLMs (Figure 7B), indicating the major role of UGT2B17 in 11β-MNT glucuronidation. Moreover, a stronger correlation was observed between UGT2B17 abundance and 11β-MNTG (Figure 7C; R^2^ > 0.99; *p*-value= 0.05) in the HH incubation. The evidence confirmed the primary role of UGT2B17 in 11β-MNT glucuronidation.

## 4. Discussion

The first-pass metabolism of drugs is one of the major contributors towards poor and variable bioavailability. In particular, drug substrates that are metabolized by intestinal enzymes (e.g., CYP3A4, UGT1A10, and UGT2B17) are prone to high metabolic intestinal extraction before entering the portal vein after oral absorption [12]. Drugs such as nifedipine, verapamil, raloxifene, testosterone, and MK-7246 are a few examples that undergo extensive intestinal first-pass metabolism, which results in their variable and poor bioavailability [9,13]. UGT2B17 exhibits a high degree of variability caused by a variety of genetic and non-genetic factors [14]. Although UGT2B17 is expressed in the liver, its abundance is 5-fold higher in the intestine than in the liver [15]. 11β-MNT, a 17β-hydroxy steroid (structurally similar to testosterone and DMA), was studied for its metabolism by UGT2B17 to explain its poor and variable oral bioavailability. In this study, a state-of-the-art metabolomics approach was used to identify metabolic products of 11β-MNT and its two prodrugs, 11β-MNTDC and 11β-MNTU in the in vitro incubations of HHs, human intestinal and liver S9 fractions, and recombinant UGT isoforms. We identified that 11β-MNT undergoes metabolism into two major metabolites, dehydrogenated 11β-MNT and 11β-MNTG. The data presented here also confirmed that polymorphic UGT2B17 plays the primary role in 11β-MNTG formation.

Previous clinical studies have demonstrated that a daily oral administration of 11β-MNTDC resulted in the 13,171 ng·h/mL (day 1) and 18975 ng·h/mL (day 28) area under the plasma concentration-time curve (AUC0–24h) of 11β-MNTDC and 63.9 ng·h/mL (day 1) and 122.6 ng·h/mL (day 28) AUC0–24h of 11β-MNT, thus resulting in an 11β-MNT/11β-MNTDC ratio of <1% [16]. However, the mechanisms of the poor bioavailability of the active 11β-MNT are unknown. We recently studied the metabolism of another 17β-hydroxy steroid, DMAU, and discovered that the polymorphic UGT2B17 is involved in its first-pass metabolism [5]. Similar to DMAU, this present study confirmed that UGT2B17 is indeed the major enzyme that plays a critical role in the intestinal metabolism of 11β-MNT. While the dehydrogenation product (M7) was the major metabolite in hepatic in vitro systems, the glucuronide product (M4) of 11β-MNT and its prodrugs were prominent in the intestinal fractions. This is consistent with our previously reported observation of intestinal abundance of UGT2B17 [17]. Moreover, one of the HH donors with a high expression of UGT2B17 showed greater levels of 11β-MNTG as compared to the dehydrogenation product. However, when the active 11β-MNT was incubated in human liver S9 at a higher concentration (50 µM), the dehydrogenation product was more prominent and likely because of the saturation of the UGT2B17 pathway. More importantly, the formation of dehydrogenated 11β-MNT was consistent relative to the parent compound in the incubations of both the active metabolite and prodrugs in the intestinal and liver S9 fractions, suggesting that the effect of the levels of the dehydrogenase enzyme(s) involved in the metabolite formation are constant across HH donors. In contrast, consistent with the high variability of UGT2B17 [14], a significantly variable formation of 11β-MNTG was observed. This indicates that although both major pathways may contribute to the poor bioavailability of the prodrugs, UGT2B17 is the primary mechanism responsible for the variable bioavailability. Although our reaction phenotyping data found a potentially minor role for other UGT isoforms in 11β-MNT metabolism, the strong correlation of UGT2B17 abundance and glucuronide metabolite levels in HH samples ruled out any possibility of a major contribution of non-UGT2B17 isoforms. 

The minor pathways of 11β-MNT metabolism identified here include glutathione conjugation, oxidation or hydroxylation, and glycosylation in HH incubations. In particular, oxidation or hydroxylation led to four distinct signals indicating that there are four different sites of possible oxidation. The absence of these metabolites in both human intestinal and liver S9 fractions in the absence of the cofactor, NADPH, suggests that these products are likely mediated by cytochrome P450 enzymes. 

There are several examples of the role of UGT2B17 in the poor and variable bioavailability of drugs. For example, the retrospective analysis of MK-7246 clinical PK data revealed that the subjects with a UGT2B17 gene deletion exhibit a 25-fold higher AUC and 82-fold greater maximum concentration of the drug (C_max_) [18]. Likewise, the population PK investigation of the recently approved anti-cancer drug belzutifan revealed that the dual UGT2B17 and CYP2C19 poor metabolizers are expected to show a 3.2-fold AUC [19], which could lead to adverse events. Moreover, our lab has recently found other substrates of UGT2B17 such as diclofenac, losartan, vorinostat, valproic acid, thyroxin, and tolcapone [20], which can also undergo extensive and variable intestinal metabolism. To address this challenge, we proposed two potential approaches [5] to increase bioavailability and reduce variability. First, subjects can be stratified using two approaches: (i) genotyping for copy number variation or (ii) utilization of the phenotypic biomarker, TG/AG, [21] to prospectively predict variability in the PK of 11β-MNTDC. Second, an inhibitor of UGT2B17 can be leveraged to reduce the intestinal first-pass metabolism of 11β-MNT prodrugs. However, there would likely be a need for a dual inhibitor of the UGT2B17 and dehydrogenase enzyme to increase the overall bioavailability of 11β-MNTDC. However, this approach is not an ideal method for contraceptives, as they are used in the long-term and could potentially lead to drug–drug interactions (DDIs). Furthermore, it is worth mentioning that there is a similar approach that is utilized to boost the bioavailability of anti-viral drugs such as nirmatrelvir for COVID-19 (Paxlovid) and several anti-HIV drugs, where ritonavir is used as a CYP3A inhibitor [22]. Moreover, to design a safe and effective clinical trial for UGT2B17 substrates, it will be important to utilize physiologically based pharmacokinetic (PBPK) modeling that accounts for drug and physiology-related parameters alongside the variability in UGT2B17 abundance, to assess the effect of UGT2B17 on the PK of 11β-MNTDC [23].

Our study suggests that the prodrug strategy is not able to bypass intestinal metabolism due to the spontaneous activation of 11β-MNTDC to the active form in the intestine. Although apparent metabolism rates of 11β-MNTDC and 11β-MNTU were slower compared to 11β-MNT alone, this effect is likely due to the poor solubility of the prodrugs. In the clinic, oral administration of 11β-MNTDC is facilitated by the co-administration of the drug with a fatty meal [24]. 

We predict that the variability in metabolism is small in the case of non-oral administration of the prodrugs. Approaches such as developing and clinically accessing 11β-MNT and its prodrugs via non-oral routes (e.g., intramuscular/subcutaneous, intravenous, sublingual, and buccal) may enable the drug to bypass intestinal and hepatic metabolism and enter the bloodstream directly.

One of the limitations of this study is that we did not identify the enzyme(s) responsible for the formation of the dehydrogenation product and other minor metabolites (formed < 2% of 11β-MNT) due to the lack of recombinant enzymes for these pathways. Nevertheless, we confirmed that dehydrogenation is relatively constant across the samples and will not be the cause of the variable bioavailability. The trend of dehydrogenated 11β-MNT formation was similar in HHs and human liver S9 fractions. Because no cofactor (NADPH) was added in the S9 fraction incubations, the involvement of Cytochrome P450s (CYPs) in dehydrogenated 11β-MNT formation was ruled out. Like testosterone, more than one hydroxysteroid dehydrogenases (HSDs) enzyme can be involved in the dehydrogenation of 11β-MNT. Based on the correlation between the dehydrogenated 11β-MNT formation and the protein abundance of HSDs in 23 individual HLMs [10], result suggests that HSD17B13, HSD17B2, HSD17B10, and HSD11B1 are likely involved in the dehydrogenation, which can be characterized in future studies with the availability of the respective recombinant enzymes. Another limitation of this work is the lack of access to human enterocytes, an equivalent system to HHs for studying complete intestinal metabolism. Nevertheless, the S9 fraction represents a subcellular fraction that expresses UGTs as well as HSDs, which are relevant in 11β-MNT metabolism in the presence of UDPGA. In this study, we have used the S9 fraction which possesses both cytosolic as well as membrane-bound enzymes to address this challenge. Moreover, enzymes involved in the conversion of the prodrug (11β-MNTDC) to the active drug (11β-MNT) will be the scope of future studies. Our metabolite data relies on MS intensity due to the lack of external standards for each metabolite identified, however, since ionization could vary, an absolute comparison cannot be confirmed.

In summary, we performed an in vitro investigation to determine the potential biotransformation products of 11β-MNT using a novel metabolomics strategy. The results of this study revealed the formation of two major metabolites, namely dehydrogenated and glucuronidated metabolites of 11β-MNT. The major participation of the polymorphic intestinal UGT2B17 enzyme in the glucuronidation of 11β-MNT elucidates the potential mechanisms of interindividual variability in 11β-MNT PK and efficacy. It is essential to consider this aspect for designing safe and effective clinical trials of 11β-MNT prodrugs. The information from the identified metabolites can be leveraged to design better analogs of 11β-MNT to address the poor and variable bioavailability of the drug. The role of UGT2B17 in the variable metabolism of 11β-MNT further highlights the importance of screening new drug entities for UGT2B17-mediated metabolism liability early in the drug development process to avoid adverse clinical outcomes such as variable PK and safety.

## Data Availability

The authors declare that all the data supporting the findings of this study are available within the paper and its Supplementary Materials.

## Supplementary Materials

The following supporting information can be downloaded at: https://www.mdpi.com/article/10.3390/pharmaceutics16081032/s1, Table S1: Optimized XCMS-based screening criteria for metabolite identification of 11β-MNT in human hepatocytes; Table S2: UGT2B17 abundance and demographic information of cryopreserved human hepatocytes; Table S3: LC-HRMS data for seven identified metabolites of 11β-MNT in human hepatocytes; Table S4: LC-MS/MS fragmentation details of 11β-MNT (50 µM) and its metabolites in human hepatocytes; Figure S1: Box and Whisker plot representing 11β-MNT and its metabolites (M1, M2a, M2b, M2c, M2d, M3, M5 and M6) showing signals in the treatment group (50 µM of 11β-MNT) compared to the control in human hepatocytes. SG: glutathione-conjugate of 11β-MNT and OH: hydroxy group; Figure S2: HRMS spectra of fragmentation of 11β-MNT (50 µM) and its metabolites in human hepatocytes; Figure S3: Quality control parameters, i.e., injection precision (A), linearity (B), sample evaporation/stability (C) for 11β-MNT and its prodrugs along with deuterated internal standards.

## Abbreviations

11β-MNT	11β-Methyl-19-nortestosterone
11β-MNTDC	11β-Methyl-19-nortestosterone dodecylcarbonate
11β-MNTU	11β-Methyl-19-nortestosterone undecanoate
11β-MNTG	11β-Methyl-19-nortestosterone-glucuronide
UGT2B17	UDP glucuronosyltransferase 2B17
HHs	Human hepatocytes
LC-MS/MS	Liquid chromatography with tandem mass spectrometry
