# Supplementary material for: Identification of the Biotransformation Pathways of a Potential Oral Male Contraceptive, 11β-Methyl-19-Nortestosterone (11β-MNT) and Its Prodrugs: An In Vitro Study Highlights the Contribution of Polymorphic Intestinal UGT2B17
Source: Pharmaceutics. 2024 Aug 2;16(8):1032. doi: 10.3390/pharmaceutics16081032 (PMC11360557; doi:10.3390/pharmaceutics16081032)
Supplement: Supplementary file 1 [file pharmaceutics-16-01032-s001.zip › pharmaceutics-3073378-supplementary.pdf]

# Identification of the Biotransformation Pathways of a Potential Oral Male Contraceptive, 11β-Methyl-19-Nortestosterone (11β-MNT) and Its Prodrugs: An In Vitro Study Highlights the Contribution of Polymorphic Intestinal UGT2B17

**Namrata Bachhav [1], Dilip Kumar Singh [1], Diana L. Blithe [2], Min S. Lee [2] and Bhagwat Prasad [1,*]**

[1] College of Pharmacy and Pharmaceutical Sciences, Washington State University (WSU), Spokane, WA 99202, USA
[2] Contraceptive Development Program, Division of Population Health Research, Eunice Kennedy Shriver National Institute of Child Health and Human Development, National Institutes of Health, 1 Center Dr , Bethesda, MD 20892, USA
* Correspondence: bhagwat.prasad@wsu.edu; Tel.: +1-(509)-358-7739; Fax: +1-(509)-368-6561

## SUPPLEMENTARY LEGENDS

**Table S1:** Optimized XCMS-based screening criteria for metabolite identification of 11β-MNT in human hepatocytes

**Table S2:** UGT2B17 abundance and demographic information of Cryopreserved human hepatocytes

**Table S3:** LC-HRMS data for seven identified metabolites of 11β-MNT in human hepatocytes

**Table S4**: LC-MS/MS fragmentation details of 11β-MNT (50 µM) and its metabolites in human hepatocytes

**Figure S1**. Box and Whisker plot representing 11β-MNT and its metabolites (M1, M2a, M2b, M2c, M2d, M3, M5 and M6) showing signals in the treatment group (50 µM of 11β-MNT) compared to the control in human hepatocytes. SG: glutathione-conjugate of 11β-MNT and OH: hydroxy group

**Figure S2:** HRMS spectra of fragmentation of 11β-MNT (50 µM) and its metabolites in human hepatocytes

**Figure S3.** Quality control parameters, i.e., injection precision (A), linearity (B), sample evaporation/stability (C) for 11β-MNT and its prodrugs along with deuterated internal standards.

**Table S1:** Optimized XCMS-based screening criteria for metabolite identification of 11β-MNT in human hepatocytes

| Criteria | m/z features |
|---|---|
| Total detected features | 7414 |
| Elevated features (>300K MS intensity, >5-fold change and p-value<0.05) | 231 |
| Features with m/z 200 to m/z 700 and MDF of -150 to +150 mDa | 167 |
| Features matching with the probable theoretical structures | 23 |
| Metabolic reactions possible with mass error <2 ppm | 10 |

**Table S2**: UGT2B17 abundance and demographic information of Cryopreserved human hepatocytes

| Human Hepatocytes (HH) | Sex | Age (Y) | Abundance of UGT2B17 (pmol/mg protein) |
|---|---|---|---|
| HH1 | Female | 54 | 0 |
| HH2 | Male | 63 | 0 |
| HH3 | Male | 50 | 12 |
| HH4 | Female | 34 | 6.8 |

**Table S3**: LC-HRMS data for seven identified metabolites of 11β-MNT in human hepatocytes

| Metabolite | Chemical formula | Theoretical mass (m/z) | Experimental mass (m/z) | Mass error (ppm) | RT (min) |
|---|---|---|---|---|---|
| Parent | $C_{19}H_{28}O_2$ | 289.2162 | 289.2157 | 1.7 | 21.6 |
| M1 | $C_{29}H_{44}N_3O_8S$ | 594.2844 | 594.2843 | 0.2 | 13.6 |
| M2 (a-d) | $C_{19}H_{29}O_3$ | 305.2111 | 305.2108 | 0.9 | 15.1 |
| M3 | $C_{19}H_{27}O_3$ | 303.1954 | 303.1951 | 1.0 | 16.8 |
| M4 | $C_{25}H_{34}O_7$ | 465.2483 | 465.2476 | 1.4 | 17.2 |
| M5 | $C_{25}H_{39}O_7$ | 451.2690 | 451.2687 | 0.8 | 19.4 |
| M6 | $C_{25}H_{39}O_8$ | 467.2639 | 467.2636 | 0.6 | 19.7 |
| M7 | $C_{19}H_{26}O_2$ | 287.2005 | 287.1999 | 1.9 | 23.0 |

**Table S4: LC-MS/MS fragmentation details of 11β-MNT (50 µM) and its metabolites in human hepatocytes**

| Metabolite | Exact mass | Experimental mass | Mass error (ppm) | Chemical formula |
|---|---|---|---|---|
| 11β-MNT | 289.2162 | 289.2157 | 1.7 | $C_{19}H_{27}O$ |
| | 253.1956 | 253.1948 | 3.2 | $C1_{9}H_{25}$ |
| | 109.0653 | 109.0650 | 2.8 | $C_7H_9O$ |
| | 95.0860 | 95.0860 | 0.0 | $C_7H_{11}$ |
| M1 | 594.2843 | 594.2844 | -0.2 | $C_{29}H_{44}N_3O_8S$ |
| | 447.2312 | 447.2305 | 1.6 | $C_{24}H_{35}N_2O_4S$ |
| | 362.2148 | 362.2147 | 0.3 | $C_{21}H_{32}NO_2S$ |
| | 287.2005 | 287.2007 | -0.7 | $C_{19}H_{27}O_2$ |
| M2-a | 305.2111 | 305.2110 | 0.3 | $C_{19}H_{29}O_3$ |
| | 287.2005 | 287.2003 | 0.7 | $C_{19}H_{27}O_2$ |
| | 251.1794 | 251.1795 | -0.4 | $C_{19}H_{23}$ |
| | 215.1435 | 215.1430 | 2.3 | $C_{15}H_{19}O$ |
| | 109.0653 | 109.0651 | 1.8 | $C_7H_9O$ |
| M2-b | 305.2111 | 305.2109 | 0.7 | $C_{19}H_{29}O_3$ |
| | 287.2005 | 287.2003 | 0.7 | $C_{19}H_{27}O_2$ |
| | 269.1899 | 269.1898 | 0.4 | $C_{19}H_{25}O$ |
| | 251.1794 | 251.1793 | 0.4 | $C_{19}H_{23}$ |
| | 241.1956 | 241.1951 | 2.1 | $C_{18}H_{25}$ |
| | 215.1435 | 215.1430 | 2.3 | $C_{15}H_{19}O$ |
| | 159.1172 | 159.1169 | 1.9 | $C_{12}H_{15}$ |
| M2-c | 305.2111 | 305.2111 | 0.0 | $C_{19}H_{29}O_3$ |
| | 287.2005 | 287.2004 | 0.3 | $C_{19}H_{27}O_2$ |
| | 269.1899 | 269.1898 | 0.4 | $C_{19}H_{25}O$ |
| | 251.1794 | 251.1795 | -0.4 | $C_{19}H_{23}$ |
| M2-d | 305.2111 | 305.2108 | 1.0 | $C_{19}H_{29}O_3$ |
| | 287.2005 | 287.2011 | -2.1 | $C_{19}H_{27}O_2$ |
| | 269.1899 | 269.1897 | 0.7 | $C_{19}H_{25}O$ |
| | 251.1794 | 251.1796 | -0.8 | $C_{19}H_{23}$ |
| | 111.8157 | 109.0650 | 2.7 | $C_7H_9O$ |
| M3 | 303.1954 | 303.1951 | 1.0 | $C_{19}H_{27}O_3$ |
| | 285.1849 | 285.1845 | 1.4 | $C_{19}H_{25}O_2$ |
| | 267.1743 | 267.1740 | 1.1 | $C_{19}H_{23}O$ |
| | 227.1430 | 227.1428 | 0.9 | $C_{16}H_{19}O$ |
| | 209.1324 | 209.1323 | 0.5 | $C_{16}H_{17}$ |
| | 157.1011 | 157.1005 | 3.8 | $C_{12}H_{13}$ |
| | 97.0647 | 97.0651 | -4.1 | $C_6H_9O$ |
| M4 | 465.2483 | 465.2479 | 0.9 | $C_{25}H_{34}O_7$ |
| | 289.2162 | 289.2159 | 1.0 | $C_{19}H_{29}O_2$ |
| | 271.2056 | 271.2053 | 1.1 | $C_{19}H_{27}O$ |
| | 253.1950 | 253.1948 | 0.8 | $C_{19}H_{25}$ |
| | 159.1168 | 159.1167 | 0.6 | $C_{12}H_{15}$ |
| | 113.0233 | 113.0235 | -1.8 | $C_5H_5O_3$ |
| | 109.0647 | 109.0650 | -2.8 | $C_7H_9O$ |
| | 85.0284 | 85.0288 | -4.7 | $C_4H_5O_2$ |
| M5 | 451.2690 | 451.2687 | 0.7 | $C_{25}H_{39}O_7$ |
| | 257.2263 | 257.2263 | 0.0 | $C_{19}H_{29}$ |
| M6 | 467.2639 | 467.2636 | 0.6 | $C_{25}H_{39}O_8$ |
| | 163.0753 | 163.0752 | 0.6 | $C_{10}H_{11}O_2$ |
| M7 | 287.2005 | 287.2001 | 1.4 | $C_{19}H_{26}O_2$ |
| | 269.1899 | 269.1897 | 0.7 | $C_{19}H_{25}O$ |
| | 251.1794 | 251.1793 | 0.4 | $C_{19}H_{23}$ |
| | 211.1481 | 211.1480 | 0.5 | $C_{16}H_{19}$ |
| | 159.1168 | 159.1158 | 6.3 | $C_{12}H_{15}$ |
| | 109.0647 | 109.0650 | -2.8 | $C_7H_9O$ |

**Figure S1**. Box and Whisker plot representing 11β-MNT and its metabolites (M1, M2a, M2b, M2c, M2d, M3, M5 and M6) showing signals in the treatment group (50 µM of 11β-MNT) compared to the control in human hepatocytes. SG: glutathione-conjugate of 11β-MNT and OH: hydroxy group

**11β-MNT**
**(m/z 289.2162)**

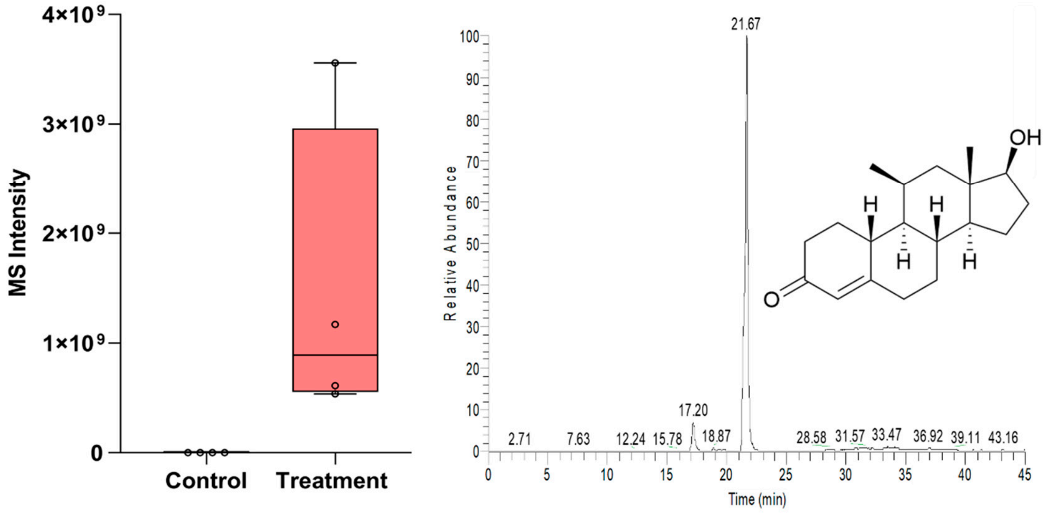


**M1**
**(m/z 594.2844)**

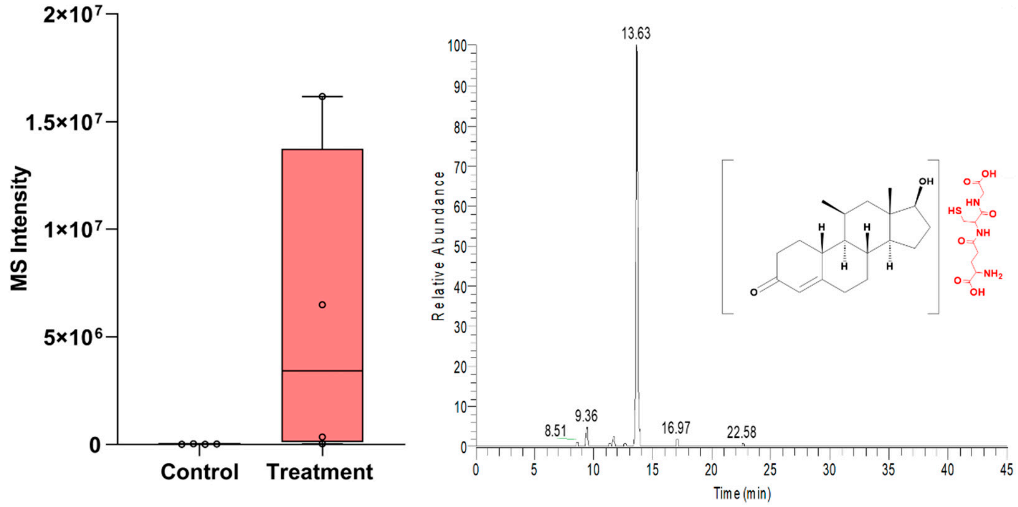

**M2 (a-d)**
**(m/z 305.2111)**

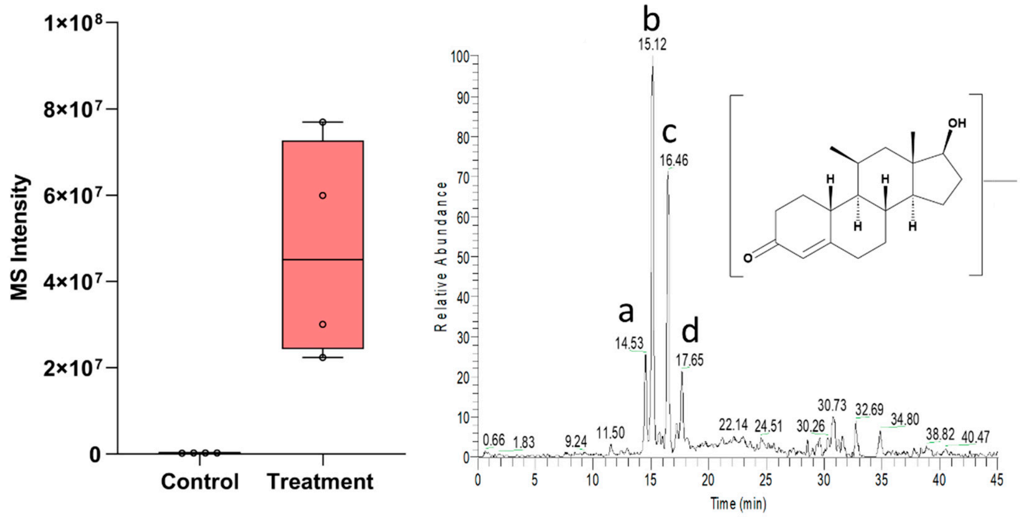


**M3**
**(m/z 303.1954)**

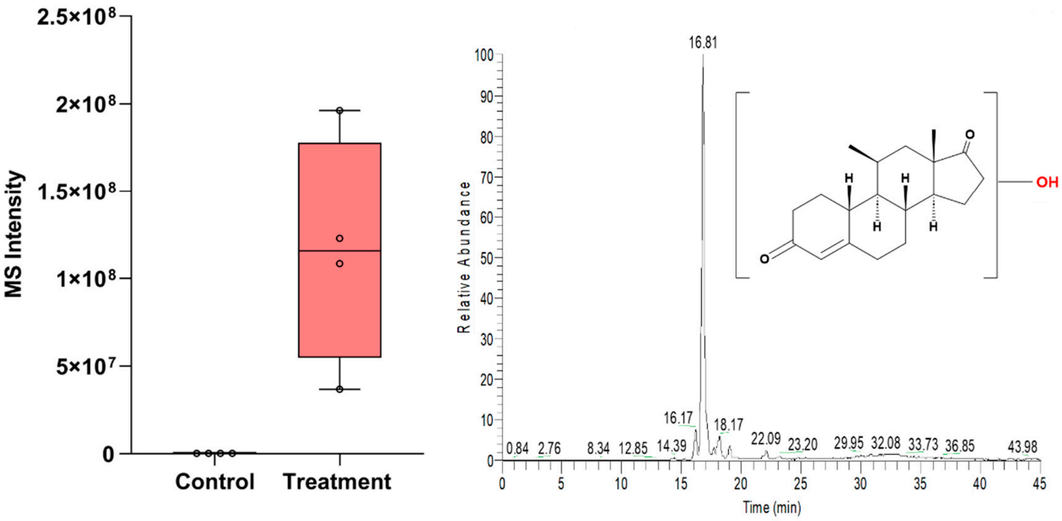

**M5**
**(m/z 451.2690)**

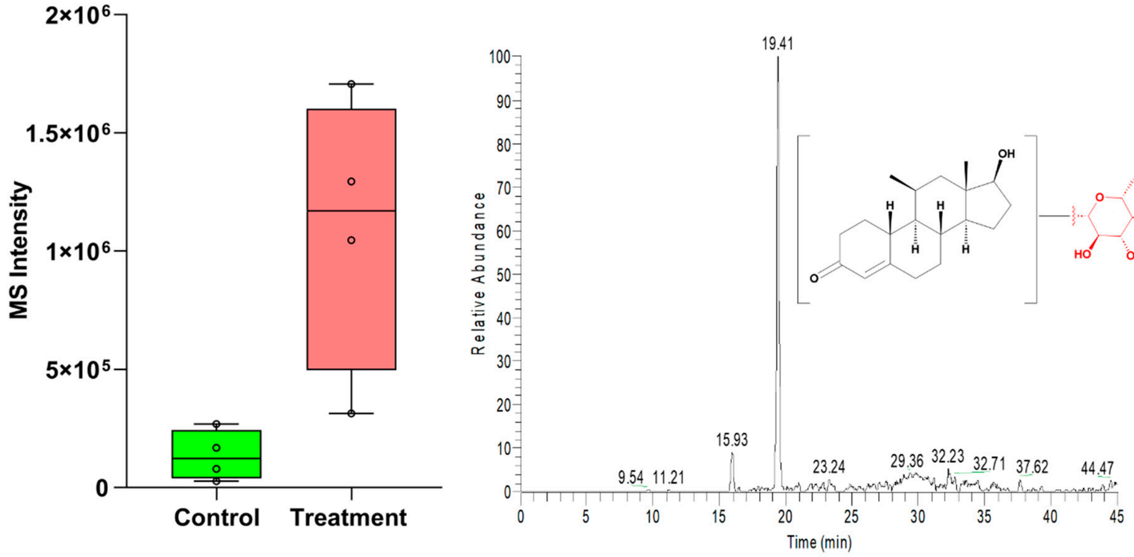


**M6**
**(m/z 467.2639)**

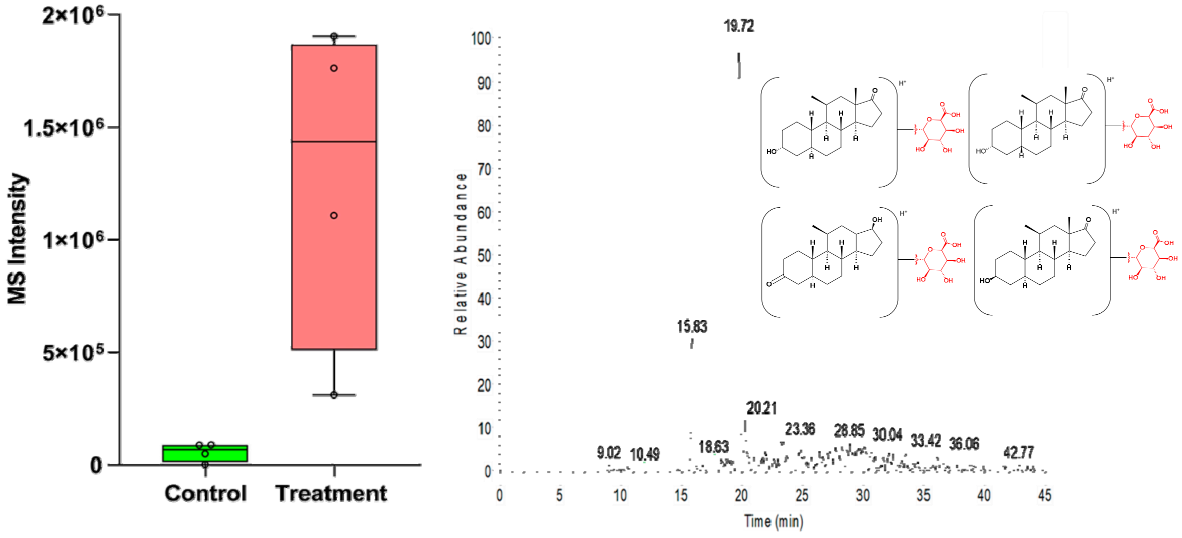

**Figure S2.** HRMS spectra of fragmentation of 11β-MNT (50 µM) and its metabolites in human hepatocytes

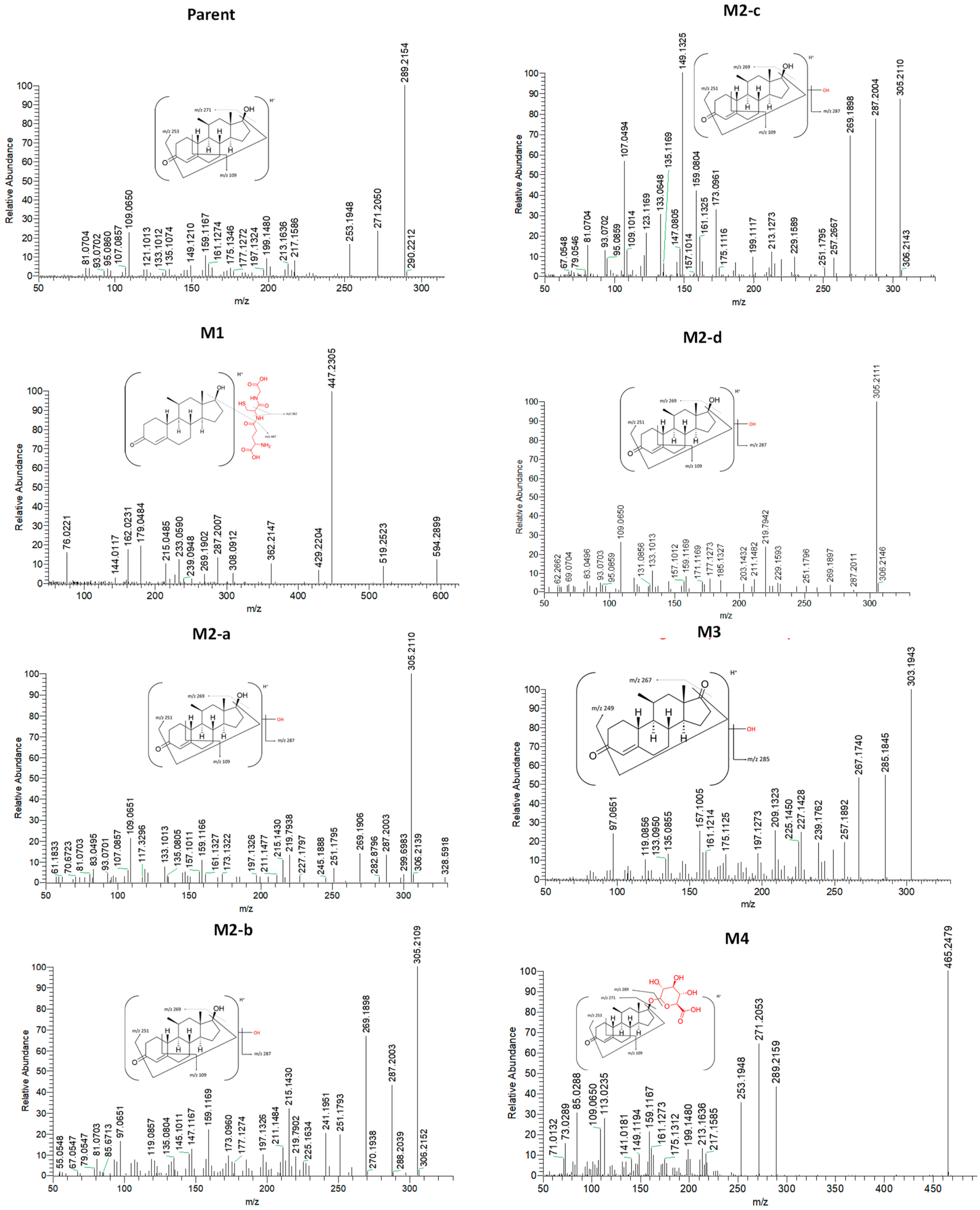

**M5**

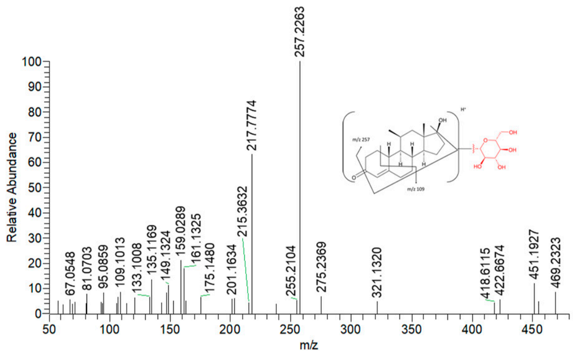
Relative Abundance
m/z
67.0548
81.0703
95.0859
109.1013
133.1008
135.1169
149.1324
159.0289
161.1325
175.1480
201.1634
215.3632
217.7774
255.2104
257.2263
275.2369
321.1320
418.6115
422.6674
451.1927
469.2323

**M7**

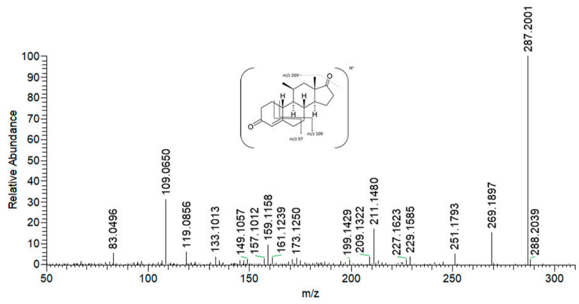
Relative Abundance
m/z
83.0496
109.0650
119.0856
133.1013
149.1057
157.1012
159.1158
161.1239
173.1250
199.1429
209.1322
211.1480
227.1623
229.1585
251.1793
269.1897
287.2001
288.2039

**M6**

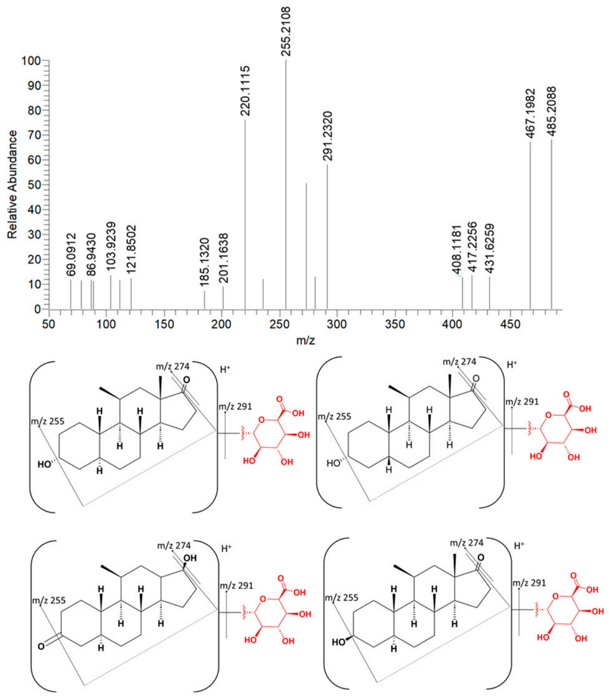
Relative Abundance
m/z
69.0912
86.9430
103.9239
121.8502
185.1320
201.1638
220.1115
255.2108
291.2320
408.1181
417.2256
431.6259
467.1982
485.2088
m/z 274
m/z 255
m/z 291
H⁺

**Figure S3.** Quality control parameters, i.e., injection precision (A), linearity (B), sample evaporation/stability (C) for 11β-MNT and its prodrugs along with deuterated internal standards.

A

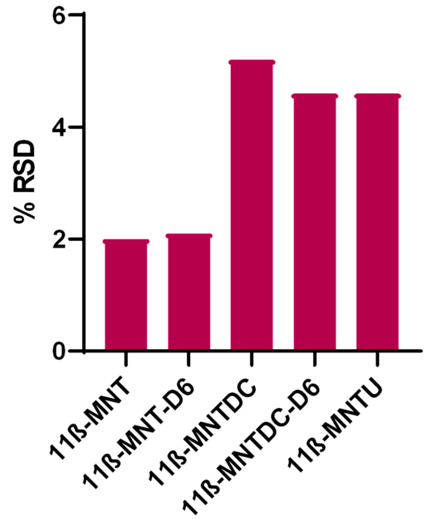


B

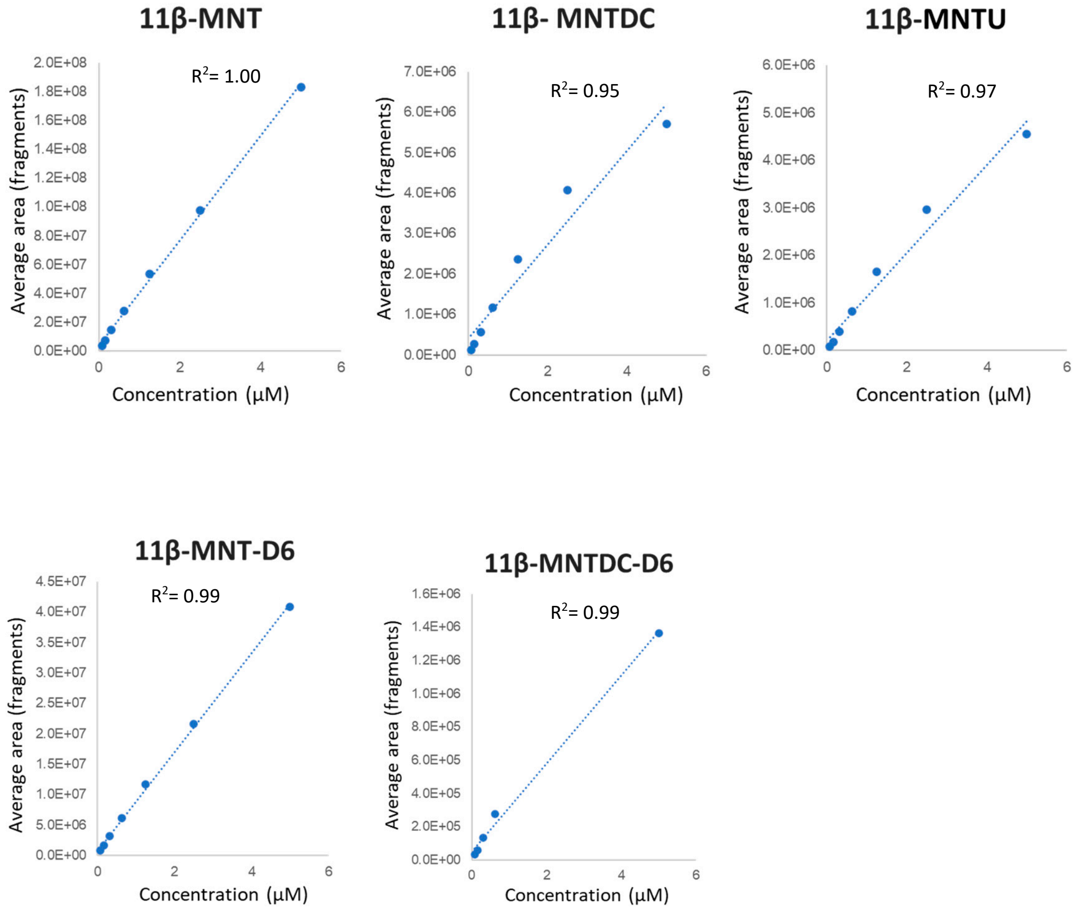

**C**

| Compound name | % Change at 18 hour without normalization | % Change at 18 hour with normalization |
|---|---|---|
| 11β-MNT | 21.8 | 0.3 |
| 11β-MNTDC | 12.2 | -0.7 |
| 11β-MNTU* | 18.0 | 4.4* |
| 11β-MNT-D6 | 21.5 | Not applicable |
| 11β-MNTDC-D6 | 13.0 | Not applicable |

* 11β-MNTU normalized using 11β-MNTDC-D6

$$\%\ \text{Change with normalization} = \frac{\text{Average normalized area ratio at 18 hour} - \text{Average normalized area ratio at 0 hour}}{\text{Average normalized area ratio at 0 hour}} * 100$$
